# Integrating early child development into an existing health and nutrition program: evidence from a cluster-randomized controlled trial

**DOI:** 10.1186/s12889-024-20149-w

**Published:** 2024-09-27

**Authors:** Caitlin Hemlock, Emanuela Galasso, Ann M. Weber, Tsirery Christian Randriamiarisoa, Mathilde Col, Maria Dieci, Lisy Ratsifandrihamanana, Lia C.H. Fernald

**Affiliations:** 1 School of Public Health, University of California Berkeley, Berkeley, CA USA; 2 Development Research Group, The World Bank, Washington, DC USA; 3 School of Public Health, University of Nevada Reno, Reno, NV USA; 4 Department of Philosophy, University of Antananarivo, Antananarivo, Madagascar; 5 Paris School of Economics, Paris, France; 6 University of Bordeaux, Bordeaux, France; 7 Department of Health Policy and Management, Rollins School of Public Health, Emory University, Atlanta, GA USA; 8 Centre Médico-Educatif ‘Les Orchidées Blanches’, Antananarivo, Madagascar; 9 School of Public Health, University of Washington, Seattle, WA, USA

**Keywords:** Early child development, Early stimulation, Implementation science, Program take-up, Play materials, Program scaling, Madagascar

## Abstract

**Introduction:**

In low-resource settings, introducing child health programs into community services may compete for caregiver time. We analyzed the impact of a new early childhood development (ECD) program in rural Madagascar on family attendance at other health services and of adding at-home play materials on program attendance.

**Methods:**

We randomized 75 communities where community health workers (CHWs) implement an existing child health and nutrition program (Projet d’Amélioration des Résultats Nutritionnels or PARN), the status quo. We offered two 6-month cycles of 12 ECD sessions to eligible caregiver-child dyads (6–30 months) in 25 sites [T]; we added take-home play materials in Cycle 2 to 25 sites [T+]. We used differences-in-differences with administrative data to analyze the effect of offering ECD sessions on monthly PARN attendance (T+/T vs. C) among age-eligible children and the impact of toy boxes/libraries on monthly ECD session attendance (T + vs. T). We used random intercept models to analyze characteristics associated with program registration.

**Results:**

We analyzed data for 9,408 dyads; 30% and 32% registered for the program in Cycle 1 and 2 (respectively). On average, CHWs delivered 11.4 sessions (SD: 1.5). Children from wealthier households who already attended PARN sessions were more likely to register, and we found no effect of T or T + on PARN attendance. Adding play materials did not affect monthly ECD session attendance. Children from more populated sites were less likely to participate in both ECD and PARN sessions.

**Conclusions:**

Integrating new services for ECD into the health system was feasible and did not reduce dyad participation in existing services. Investment in health services in more populated areas is needed to provide coverage to all eligible children. Novel strategies should be explored to engage the most vulnerable children in new and existing health services.

**Trial registration:**

AEA Social Science Registry (AEARCTR-0004704) on November 15, 2019 (prospective registration) and ClinicalTrials.gov (NCT05129696) on November 22, 2021 (retrospective registration).

**Supplementary Information:**

The online version contains supplementary material available at 10.1186/s12889-024-20149-w.

## Background

Children living in poverty or experiencing malnutrition risk not reaching their full developmental potential [1], leading to reduced cognitive capacity, poorer schooling outcomes, and reduced participation in labor markets [2, 3]. Early child development (ECD) interventions in low-resource settings, typically parenting programs delivered through intensive 1-on-1 home visits [4], focus on nurturing care and stimulating activities. These programs have been effective in small-scale studies [5], but few have been successful at scale [6]. Thus, recent work has explored how to deliver effective programs at scale and overcome challenges such as limited frontline worker capacity, time- and resource-constrained caregivers, and variation in program quality and target population coverage [7, 8].

Recently, some programs were adapted to group-based delivery, bringing together caregivers and children in a central location [9–12]. A benefit of this is decreasing the burden on delivery agents, usually frontline workers with competing demands and priorities, by delivering messages about ECD and stimulating activities to multiple caregivers at once. Group-based delivery also creates a community support network, which can improve maternal mental health, a known factor related to child cognitive development [1]. Delivering ECD-promoting interventions in groups is a feasible alternative implementation strategy to increase the proportion of the population reached and maximize coverage, with comparable effectiveness to home visits at a lower cost [13]. Implementation research focused on the best mechanisms for delivery, such as studying fidelity of delivery, acceptability by the program’s target population, and program coverage is essential to scaling effective interventions [14].

A common approach to scaling ECD interventions involves integrating ECD promotion into existing health infrastructure [7, 8]. In Madagascar, where 40% of children are stunted and 80% live in extreme poverty [15], the government has implemented a maternal and child health and nutrition program (Projet d’Amélioration des Résultats Nutritionnels, or PARN) nationwide, delivered in each community by two frontline community health workers (CHWs). Over 8,000 CHWs work nationwide and are paid by the government and supervised by regional offices and local non-governmental organizations. A previous trial (MAHAY) in the same setting delivered fortnightly home visits on nutrition and early stimulation with a dedicated additional CHW [16]. Although the MAHAY intervention was carefully implemented, it failed to demonstrate significant effects of the program components on ECD outcomes. Hypothesized explanations include supply-side constraints, such as long travel distances for CHWs to perform home visits, and caregivers’ limited ability to act on behavioral messaging delivered by CHWs, potentially from a lack of time or mental capacity to participate in activities or lack of materials to implement the intervention’s suggestions regarding play.

To overcome these implementation constraints, the curriculum delivered in MAHAY home visits was adapted to a group setting (MAHAY Mikolo, “know how to take care”) and integrated into the existing PARN service delivery platform and the existing CHWs, hypothesized to leverage economies of scope. The group sessions allow for increased coverage while reducing the amount of travel spent by CHWs and strengthen the support to parenting skills with behavioral nudges and age-appropriate toys and books. Participating in an additional component of the health and nutrition program may increase the caregivers’ potential interest and knowledge, because of its novelty and supportive nudges. Conversely, it may be time-consuming for caregivers, who have competing demands on their time from household, caregiving, and work responsibilities, potentially crowding out participation in the status-quo program.

This study aimed to assess the impact of integrating new services for ECD into an existing health and nutrition program on attendance and participation in ECD, nutrition, and health activities using a cluster-randomized controlled design. Our first research question asked if offering the new ECD program would change participation in the status quo health programming (PARN) and hypothesized that the new ECD program may increase participation in PARN by caregiver-child dyads by inciting interest (crowding in) or decrease by competing for caregiver time (crowding out). Our second research question asked if adding age-appropriate toys would change attendance at the ECD sessions, and we hypothesized that attendance at ECD sessions would increase. Our secondary research question examined the heterogeneity of our primary outcomes by different characteristics, including geography, CHW education, and population size. We also analyzed post-hoc characteristics associated with take up of the program and the effect of toy boxes on sustained child attendance and parental attendance.

## Methods

### Study Design

The MAHAY Mikolo study was a multi-arm, parallel, cluster-randomized controlled trial conducted from March 2021 to February 2022 in two regions of Madagascar, Amoron’i Mania and Haute Matsiatra, which are in the central highlands; details about the study design and rationale are available in the previously published study protocol [17]. We focused on two representative regions targeted by the government PARN program (Amoron’i Mania and Haute Matsiatra), originally selected for the high prevalence of childhood malnutrition. We excluded extremely remote ones due to the study design, which necessitated monthly data collection and monitoring and were infeasible in very remote districts. From the districts of interest, we excluded sites with other ECD programs present in the community or neighboring communities, less than 40 children attending nutrition monitoring sessions to ensure sufficient demand for the program, and turnover in the non-governmental organization (NGO) contracted to supervise CHWs in the last year, to allow for quality intervention monitoring. Sites are defined as communities where PARN is delivered by two CHWs (thus CHW catchment areas).

The study protocol was approved by the Committee for Protection of Human Subjects at University of California, Berkeley (protocol number: 2019-08-12476). The trial was registered prospectively on the AEA Social Science Registry (AEARCTR-0004704) on November 15, 2019 and retrospectively on ClinicalTrials.gov (NCT05129696) on November 22, 2021. Informed consent to participate was not obtained from caregivers or parents/legal guardians of any participant under the age of sixteen. UC Berkeley Committee for Protection of Human Subjects deemed consent to participate unnecessary according to national regulations as data was administrative in nature.

## Randomization and blinding

From the eligible sites participating in the PARN program in the two regions, we used stratified randomization to account for characteristics that may be strongly related to our outcomes of interest. We used administrative, publicly available data to stratify on the following four dimensions: at least one CHW has Secondary II education or higher (nine years of basic education or more) or none, distance from the center of the site to a health center (binarized to above and below median), size of the target population of children (6–30 months old) at the site (binarized to above and below median), and region. From the 16 strata created, we randomly sampled six communities from each of the seven smaller strata or 12 communities from each of the larger nine strata to create a set of 25 sextuplets. Next, we used a random number generator to randomly draw three communities from each sextuplet and allocate them to one of the three study arms using a 1:1:1 allocation ratio (conducted by MC). This ensured that there were three backup communities in each stratum in case of refusal during recruitment or dropout at the beginning of the study. To minimize contamination, we ensured that treatment and control communities did not have the same local NGO as supervisors and replaced any control communities where a site assigned to treatment was within a 1.5-kilometer radius. More details about the stratification and the sampling are available in the study protocol [17].

The regional nutrition offices and NGO supervisors visited communities selected for the intervention arms to solicit interest and consent to participate and obtain buy-in from CHWs, who would not be compensated for additional time spent on the program but be provided quarterly phone credit as well as an in-kind gift at the end of the intervention. Thus, as indicated in the protocol, neither participants nor the intervention delivery team were blinded due to the nature of the intervention. Statistical analyses were conducted masked using a scrambled treatment assignment variable before revealing results to investigators using the true assignment variable.

## Intervention

The MAHAY Mikolo intervention consisted of age-specific ECD stimulation activities delivered by CHWs to caregiver-child dyads in a group setting. The curriculum was partially based on the parenting program Reach Up and Learn, originating from Jamaica [4] and previously adapted to the Malagasy context for home visits [16]. The curriculum was subsequently adapted to be delivered through 12 fortnightly group sessions over six months to four age groups: 6–12 months, 12–18 months, 18–24 months, and 24–30 months. CHWs delivered the activities over a 60 to 90-minute period in the nutrition and health center that hosts the national community-based nutrition and health program (usually located at the center of the site). The sessions also included behavioral nudges [18], such as a pledge to highlight the caregiver’s commitment, distribution of reminder sheets to reinforce messages learned during the sessions, and suggestions for activities to do at home in between sessions.

CHWs in treatment communities attended a two-week training session where they learned about child development, the importance of early stimulation, and the curriculum contents. After an initial in-person coaching session, the CHWs also received monthly coaching by phone, a strategy to reduce costs and provide regular assistance for scale-up. After delivering the first 6-month curriculum (Cycle 1), the CHWs received a refresher training. They also conducted additional information sessions to encourage children age-eligible for the next installment (Cycle 2) to sign up and children who attended Cycle 1 to transition to the next age group.

Both treatment arms (T and T+) received the ECD group sessions and one of the treatment arms (T+) at the beginning of the Cycle 2 added an age-specific and developmentally-appropriate toy box to each registered child, as well as a rotating set of toys and a book library with the same materials from the sessions. Caregiver-child dyads who attended a given session could borrow and take home a toy and book to use in between sessions, further incentivizing caregivers to return for the next ECD session and practice activities learned during the sessions, promoting sustained play at home. CHWs in the T + arm received additional training on these activities. The toy boxes in T + were procured centrally and delivered by the CHWs and supervisors at the beginning of Cycle 2. The materials and books from the sessions in both T and T + were produced and maintained by a team of trained youth volunteers, in partnership with the status quo program.

The control group consisted of the standard of care in study sites (PARN), which is managed by Unité - Programme de Nutrition Communautaire (U-PNNC), an agency in the Office National de Nutrition Madagascar. The program consists of a package of maternal and child health and nutrition interventions, including regular growth monitoring, monthly cooking demonstrations, community-based integrated management of childhood illnesses, referrals to health centers for services, such as vaccinations and treatment of acute malnutrition, and home visits as needed. CHWs in all treatment arms received a monthly salary for delivering the PARN program. CHWs and supervisors in treated communities were compensated for the extra time spent on delivering the ECD sessions, with in-kind gifts provided at the end of the pilot.

Families with children aged 6–30 months in the intervention arms were invited to community mobilization and information sessions hosted at each site center and in neighborhoods within each site (called “hamlets”), where CHWs and their supervisors described the program eligibility and enrolment process, the structure and content of the sessions and the time commitment required by the caregivers. During these mobilization sessions, names of all caregivers who were interested in enrolling in the program were collected. Households or caregiver-child dyads could also register with the CHWs or NGO supervisor during the two weeks following the mobilization campaign.

The program was capped at 10 caregiver-child dyads per six-month age group per cycle, for a total of 40 spots per site, to prevent overburdening the two CHWs already tasked to deliver the status quo program. If there was more demand than could be accommodated for a given age group, CHWs assigned spots on a first come first served basis, or randomly drew 10 households from the list of interested households, with the remainder added to a potential waitlist due to the limited space in the program. We define a dyad as registered when the caregiver signed up at the mobilization campaigns and was enrolled in the ECD program.

## Participants and procedures

To determine the denominator and identify all age-eligible children in each site, CHWs updated the site censuses in February 2021 (baseline). For the study, we followed the cohort of children ascertained during the census as age-eligible for Cycle 1 (eligible = aged 6 to 30 months on March 1, 2021; Cycle runs from March to August) or Cycle 2 (eligible = 7–30 months on September 1, 2021 [children less than seven months old were excluded due to the timing of the census where a denominator was obtained]; Cycle runs from August to February) (Supplementary Fig. 1). We established six sub-cohorts of age-eligible children based on their age at the beginning of Cycle 1: A = 0 to < 6 months, B = 6 to < 12 months, C = 12 to < 18 months, D = 18 to < 24 months, E = 24 to < 30 months. The six-month age bands correspond to the age-specific ECD groups implemented in Cycle 1 and Cycle 2.

## Data collection

We used several sources of data for our analyses, which were mainly administrative. As a part of the regular monitoring and evaluation system of PARN, CHWs recorded attendance by children to health and nutrition sessions on paper registries. Attendance records were collected, entered using tablets, and uploaded to a digital data platform (CommCare, https://www.dimagi.com/commcare/) by NGO supervisors, as part of their monthly routine tasks. For the ECD group sessions, we developed an additional registry and CommCare ECD module where CHWs recorded the unique identifier for each child who attended a session, which caregiver attended the session with the child, and whether the child borrowed toys or books in the T + arm; this registry was filled in on paper by CHWs and entered into CommCare by supervisors. Due to CHW and supervisor payment issues and accessibility issues related to the pandemic, a subset of ECD session data were available only on paper, and had to be scanned and extracted later by Optical Character Recognition (OCR) by the research team. CHWs also conducted the household census as part of their yearly administrative task in their respective communities. In addition to registering new children, the CHWs were asked to collect additional information on household socioeconomic status for the purposes of the study, including maternal education, wall material, and roof material. This information was only collected on a subset of households due to difficulties collecting additional data beyond household rosters. Census data were also collected on paper and data entry was performed by the National Institute of Statistics of Madagascar. Given the administrative nature of all census and registry data, informed consent of dyads participating in health and nutrition sessions and ECD sessions was not obtained. Data were anonymized by a data manager after downloading from the server and prior to analysis by removing personal identifiers and keeping anonymous identification numbers only.

In addition to the administrative data, the study included a phone survey administered to CHWs and village key informants, conducted 10 times over the 12-month study period. CHWs were asked about their own time use, any household shocks they experienced, and details on CHW services delivered; village key informants were asked about village infrastructure, service availability, prices, and economic and weather-related shocks. CHWs in all treatment arms were provided with phone mobile credit as a compensation for survey participation. The survey data was used only to obtain socio-economic descriptive statistics on the community site and the CHWs and was not used in the analysis.

### Outcomes

Primary outcomes were assessed among children age-eligible for the ECD program and included any attendance at PARN sessions where health and nutrition activities are conducted in a given month and any attendance at an ECD session in a given month. We assessed all outcomes at the child level. We assumed that if the child identification number was not present in the registry for that month, they did not attend. We also analyzed post-hoc outcomes including registration for the cycle (or program take up), any attendance during the cycle (only monthly attendance was prespecified), and the probability of parental attendance (a parent accompanying the child to the ECD sessions compared to siblings/grandparents, neighbors).

### Statistical analysis

We performed power calculations for pairwise comparisons of 25 treatment and 25 control sites (50 sites). We calculated the minimum detectable effect (MDE) for clustered randomized trials with repeated measures using the software package Optimal Design (OD) Plus (v3.01). We assumed a minimum of 40 caregiver-child dyads in each site at each of the 12 time points given our site exclusion criteria and an ICC ranging from 0.1 to 0.3 for participation outcomes. Under these assumptions, we were powered for at 80% for MDEs ranging between 0.31 SD (ICC = 0.1) and 0.47 SD (ICC = 0.3).

We used random effects models to identify variables associated with program take up, which we defined as program registration during information sessions. We first created a null model to estimate the variance components of take up by including a random effect for site and child. Second, we added site-level stratification variables. Third, we added individual-level variables including gender, sub-cohort (Cohort A-E, see Participants and Procedures above), and number of siblings. Fourth, we added variables associated with socioeconomic status, including wall type, roof type, and years of maternal education. These were included in a separate model as they were not captured for all children due to difficulties in collecting data beyond the administrative data regularly collected.

We estimated treatment effects by analyzing outcomes of all age-eligible children in intervention and control sites regardless of enrollment in the program (intention-to-treat). We included fixed effects for strata in all models to control for our sampling design [19]. Our previously pre-specified empirical specification to test for differences in health and nutrition session attendance used treatment dummies and time fixed effects [17]. We switched to a differences-in-differences (DiD) design due to imbalance at baseline on our primary outcome (attendance at health and nutrition sessions) to control for time trends and unobservable differences between groups, and we made this change prior to analyzing the treatment effects. First, we looked at the primary outcome in the 6 months before baseline and checked the parallel trends assumption. Next, we tested the following DiD specification using a scrambled treatment ID number:

$$\begin{gathered}{Y_{ijt}} = {\beta _0} + {\beta _1}{T_j} + \beta {}_2T{ + _j} + {\beta _3}C{1_t} + {\beta _4}C{2_t} \hfill \\+ {\beta _5}{T_j}*C{1_t} + {\beta _6}{T_j}*C{2_t} + {\beta _7}T{ + _j}*C{1_t}\hfill \\ + {\beta _8}T{ + _j}*C{2_t} + {\theta _s} + {\lambda _c} + {\epsilon _{ijt}} \hfill \\ \end{gathered}$$

where Y_ijt_ is whether a child *i* in site *j* attended a health and nutrition session in a given month *t*, T_j_ and T + _j_ are dummies for treatment assignment for site *j* (control group is the comparison), C1_t_ and C2_t_ are dummies for whether month *t* was in Cycle 1 or Cycle 2, θ_s_ are strata fixed effects, λ_c_ are cohort fixed effects, and ε_ijt_ is the error term. The parameter of interest for the impact of the program in the difference in difference specification are the interaction of the treatment dummies and the cycle dummies (β_5_-β_8_), that is the outcome in treatment communities during the intervention period of Cycle 1 and Cycle 2.

We used the true treatment assignment in two main specifications: (1) including outcome data collected from one month before the intervention (equivalent in theory to our ANCOVA specification) and (2) including an additional two months of data in the pre-period as a robustness check of parallel trends.

We performed F-tests to determine the effect of the program on nutrition session attendance compared to control (joint test of H_0_: β_5_ = 0 and H_0_: β_6_ = 0), the effect of the program plus toyboxes on nutrition session attendance compared to control (joint test of H_0_: β_7_ = 0 and H_0_: β_8_ = 0), and if the addition of toyboxes affected nutrition session attendance (H_0_: β_6_ = β_8_). We also tested if there was a difference between T and T + during Cycle 1 as a negative control (H_0_: β_5_ = β_7_). We also performed two sensitivity analyses: (1) excluding Cohort A in Cycle 1 and Cohort E in Cycle 2 in the health and nutrition session attendance outcomes, since these sub-cohorts were not eligible in the respective cycles which may bias the effects towards the null and (2) controlling for observed variables that were imbalanced at baseline as a robustness check. For analyses comparing T and T + only, we only used data in Cycle 1 and Cycle 2 and among children eligible for each cycle (thus excluding Cohort A in Cycle 1 and Cohort E in Cycle 2).

We assessed for heterogeneity using a triple difference estimator and added a full set of interactions between the treatment indicators (β_5_ - β_8_ in the equation above) and a third variable of interest [20]. For health and nutrition session attendance, we tested for heterogeneity on site-level variables used for stratification (CHW education (whether at least one CHW had > = 9 years of formal education compared to none), region (Haute Matsiatra compared to Amoron’i Mania), distance to health center (above and below median), and target population size quartile. We tested for heterogeneity in the impact of toyboxes on ECD attendance by age sub-cohort (Supplementary Fig. 1).

For all models analyzing treatment effects, we used ordinary least squares regression and calculated robust standard errors clustered at the site level to correct for correlated error structures between children within sites and between months within children. All statistical analyses were performed in R version 4.2.1. Statistical code is available on github (1pt.co/lwy0m).

## Patient and public involvement

We pre-tested the curriculum extensively in a six-month pre-pilot in one community in 2018 and a one-year pilot in two communities in 2019–2020. Feedback from CHWs, caregivers and PARN program implementers was used to refine the intervention components.

## Results

Among the 1,348 communities in the PARN program in the selected regions, 1,185 were eligible for inclusion in the study. We drew a stratified sample and randomly assigned 75 communities to the three arms (Control, T, and T+). One community refused and was replaced prior to recruiting caregiver-child dyads. Among the 22,958 children in the census at the start of the study, 37 (< 0.1%) did not have birthdate information available and 13,513 (59%) were older than 30 months (Supplementary Fig. 2). Among the remaining 9,408 children, 8,067 (86%) were eligible for Cycle 1; 1,341 aged in for Cycle 2 (Cohort A), while 2,379 aged out for Cycle 2 (Cohort E) (Supplementary Fig. 1). From February 2021 to February 2022, we collected monthly data on attendance at health and nutrition activities on these 9,408 children, resulting in 112,072 monthly observations.

Given the attributes used in our stratified clustered design, the sites were balanced (Table 1). However, after the census was performed in February 2021, we found that the target population size in the control group was larger than T and T + sites and the overall size of the population was larger in T + compared to T. After reviewing our primary outcome, attendance at the health and nutrition sessions, we found imbalance across groups at baseline (Table 1), however, pre-trends were similar (Supplementary Fig. 3). In terms of the CHWs delivering the intervention, sites were balanced on CHW age, education, and years of experience.

**Table 1:** Characteristics of study sites (*n* = 75)

	Control, *n* = 25	T, *n* = 25	T+, *n* = 25
**Site characteristics during sampling (2020)**			
Distance to nearest healthcare center (km)	1.70 (0.33, 3.06)	1.75 (0.01, 3.08)	2.03 (0.63, 3.43)
Area of site (km^2^)	5 (3, 9)	7 (4, 11)	7 (4, 14)
Number of hamlets	4 (4, 7)	4 (4, 6)	5 (4, 7)
Number of children aged 6–30 months	80 (58, 91)	81 (58, 103)	83 (65, 110)
Share of children aged 6–30 months attending nutrition sessions in past two months	74% (60%, 83%)	77% (69%, 85%)	77% (70%, 82%)
**Site characteristics at baseline (2021)**			
Total population size (from survey)	1,815 (1,300, 2,267)	1,340 (1,200, 1,806)	1,578 (1,200, 3,026)
Number of children aged 6–30 months	114 (84, 131)	95 (70, 116)	103 (75, 147)
Share of children aged 6–30 months attending nutrition sessions in the past month	48% (33%, 71%)	56% (36%, 70%)	60% (47%, 69%)
**CHW characteristics (from survey)**			
Average age (yrs)	42 (37, 48)	44 (40, 49)	42 (39, 50)
Average education (yrs)	9 (8, 10)	9 (8, 10)	8 (7, 10)
Average experience (yrs)	6 (4, 8)	4 (2, 8)	6 (3, 11)

Note: Median (IQR) presented

### ECD program implementation

Ninety-five percent of planned sessions were conducted, and this was balanced across groups (Table 2). Among the children who attended at least one session in Cycle 1 and Cycle 2, 19% were not age-eligible at the time of enrollment at baseline and the month preceding the start of Cycle 2, which was balanced across groups, and the highest levels of ineligibility were in the youngest and oldest groups (Supplementary Fig. 4). Among those ineligible, 12% were two months younger (4–5 months) and 18% were two months older (30–31 months). All toy boxes with age-appropriate toys were delivered at the beginning of Cycle 2 in T + communities. Tracking the borrowing of toys and books (with check in and check out dates by item and child) resulted to be time consuming and challenging for CHWs. As a result, missing data for toy and book borrowing was high but among children with available data, a third of children borrowed books at least once, and almost half borrowed toys at least once.

**Table 2:** Implementation and descriptive characteristics of ECD sessions (treatment)

	*Cycle 1*		*Cycle 2*	
	*T*	*T+*	*T*	*T+*
Sessions conducted, n (%†)	1153 (96%)	1130 (94%)	1133 (94%)	1162 (97%)
Average number of sessions conducted per site‡, mean (range)	11.5 (4–12)	11.3 (1–12)	11.3 (4–12)	11.6 (9–12)
Average number of children in attendance per session, mean (SD)	6.9 (2.4)	6.7 (2.4)	7.0 (2.5)	7.6 (2.2)
Unique children attending any sessions	1214	1092	1103	1147
Not age-eligible	263 (22%)	165 (15%)	222 (20%)	210 (18%)
Borrowed books at least once*	-	-	-	272/858 (32%)
Borrowed toys at least once*	-	-	-	386/858 (45%)

†Out of curriculum-set 1200 sessions (25 sites x 12 sessions x 4 age groups)

‡Expected 12 sessions per age group and cycle

*Denominator includes children that attended sessions (regardless of age eligibility) with any data on toy or book borrowing

### ECD program coverage and registration

**Fig. 1:**
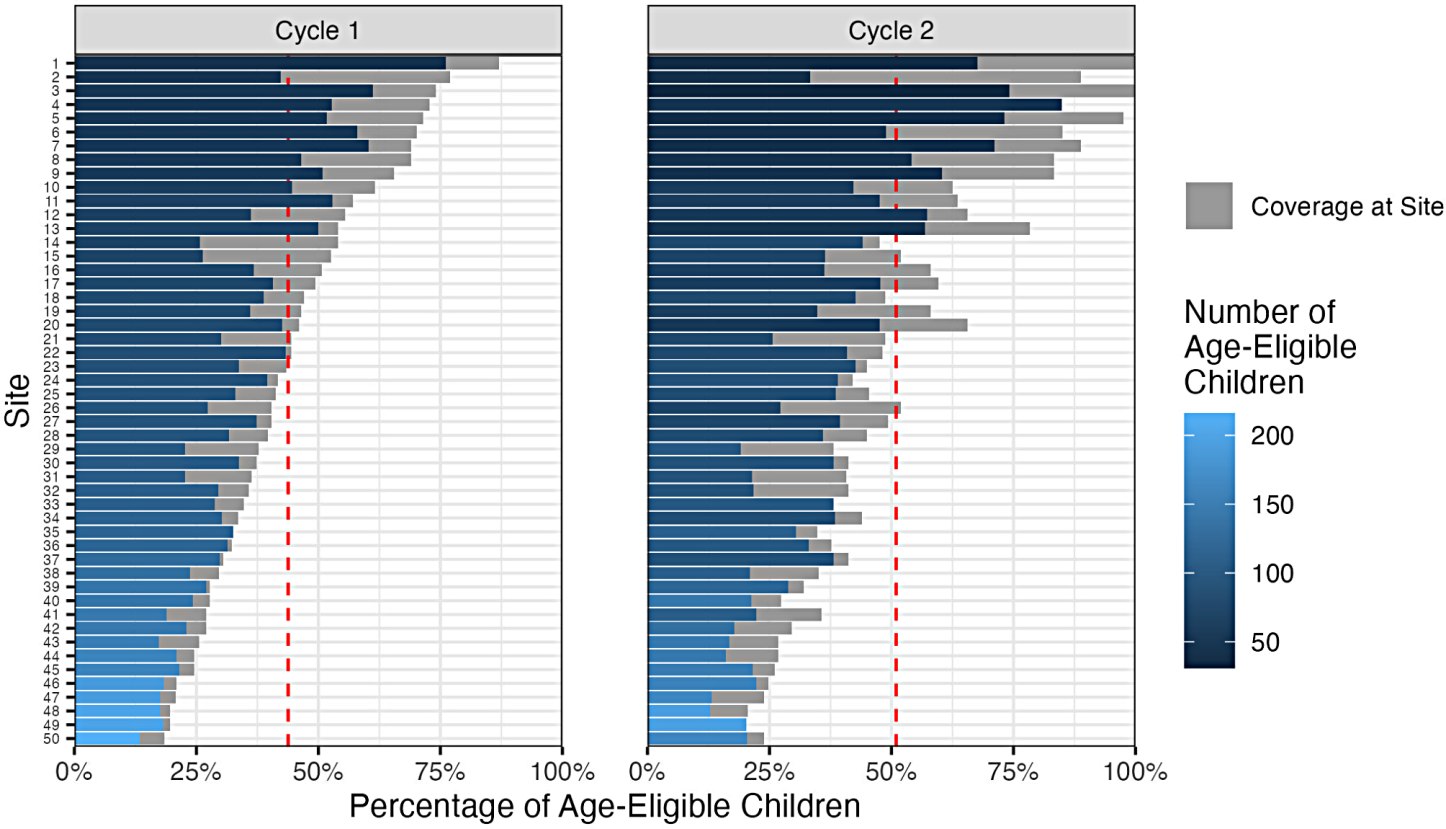
Coverage and take-up of ECD program for age-eligible children and registration, by site and cycle. Note: Colored bars represent the proportion of age-eligible children who signed up for the program. Gray bars represent available spaces in the program out of total number of age-eligible children at the site. Red dashed line is the cycle-specific overall mean coverage of spots in ECD sessions for age-eligible children

We identified 5,366 children as age eligible for Cycle 1 and 4,709 children as eligible for Cycle 2 in T and T + sites. Based on the number of available spots per cycle (10 for each of the 4 age groups = 40 spots per community and 2,000 spots total across all treatment communities), and an average population size of 100 age eligible children per community, we defined coverage as the ratio between the number of children who registered for the ECD sessions (capped at 40 per site and cycle) and the number of age-eligible children in the treatment communities. Coverage of the eligible population was constrained to be below 100% for most sites (Fig. 1), ranging from 19 to 87% in Cycle 1 (mean: 44%) to 20–100% in Cycle 2 (mean: 51%).

The overall probability of registering for sessions, after accounting for site- and child-level clustering, was 35%. After accounting for individual variables, we found no significant difference in signing up from Cycle 1 to Cycle 2 across groups (Supplementary Table 1). In line with the maximum limitation of the number of available spots to 40 per site, the probability of registering for sessions was negatively associated with the size of the age-eligible population: caregivers were 3% points less likely to sign up per 10 additional age-eligible children within the site (95% CI: -0.03, -0.04).

We also found that having previously regularly attended the PARN program was strongly associated with registering (Supplementary Table 1). Children were 6% points more likely to sign up for the program for each health and nutrition session attended in the six months preceding the start of the cycle (95% CI: 0.05, 0.06). Additionally, children who lived in sites closer to the capital city (Amoron’i Mania region) were more likely to register (0.06, 95% CI: 0.02, 0.10]) and marginally more likely in sites with more highly educated CHWs (0.04, 95% CI: -0.01, 0.08), but children in the older Cohort E were less likely to register for the program.

In the model including socioeconomic status variables, we found that wealthier households, as represented by wall materials, were more likely to register for the program (8% points [95% CI: 0, 0.16] more likely to register if wall material was made of clay and metal materials compared to plant/tree material and 12% points (95% CI: 0.03, 0.22) more likely for stone/cinder blocks). Missingness of SES variables was differential across age cohorts and treatment groups, so associations between wealth and registration should be interpreted within the population with available data (Supplementary Table 2).

### ECD session attendance and effect of toyboxes

Among the eligible children registered for the program in each cycle, most children attended the first session of the program (1,046 (65%) in Cycle 1 and 1,182 (79%) in Cycle 2). Among those who attended the first session, the average number of sessions attended was 9.1 sessions in Cycle 1 and 10.1 sessions in Cycle 2. 31% of children attended all 12 sessions in the first cycle of the program, and this increased to 41% in Cycle 2.

In our ITT analysis examining all age-eligible children in the T and T + sites, we found no effect of toyboxes on monthly ECD session attendance, comparing T + to T (Table 3). Across groups, monthly ECD session attendance was significantly lower in larger sites. Cohort E (the oldest cohort eligible in Cycle 1) was also 10% points less likely to attend, controlling for cycle and group, compared to the other cohorts. There was no differential effect of T + by sub-cohort. There was also no effect of toyboxes on parental attendance, and across treatment groups attendance at the sessions by parents decreased in the older ECD age groups (replaced by caregiver participants such as siblings, grandparents, and neighbors) (Supplementary Table 3). We did find evidence of an effect of toyboxes among children who were eligible but had not attended any sessions during Cycle 1. These children in T + sites were 4% points more likely to attend any sessions during Cycle 2 compared to T (95% CI: 0, 0.08) (Supplementary Table 3) and attended an average of 6.3 sessions (SD: 4.5 sessions). The addition of toyboxes had no effect on any attendance in Cycle 2 among children who attended sessions in Cycle 1 (i.e. children who attended any sessions in Cycle 1 were equally as likely to attend in Cycle 2 whether or not toyboxes were introduced).

**Table 3:** Effect of toyboxes on monthly attendance in ECD sessions, including heterogeneity across age cohorts

	Monthly attendance at ECD sessions (1)	Monthly attendance at ECD sessions (2)
**Cycle**		
Cycle 1	Ref.	Ref.
Cycle 2	0.01 (-0.02, 0.03)	0.03 (0.00, 0.06)
**Group**		
T	Ref.	Ref.
T+	0.02 (-0.01, 0.05)	0.00 (-0.05, 0.05)
**Cohort**		
B	Ref.	Ref.
C	0.00 (-0.02, 0.03)	0.02 (-0.03, 0.06)
D	-0.01 (-0.03, 0.02)	-0.01 (-0.05, 0.03)
E	-0.10*** (-0.12, -0.07)	-0.10*** (-0.14, -0.06)
A	0.02 (-0.01, 0.05)	0.01 (-0.04, 0.06)
Target population size (per 10)	-0.02*** (-0.03, -0.02)	-0.02*** (-0.03, -0.02)
**Group x Cycle [DiD]**		
Cycle 2 x T+	0.02 (-0.01, 0.05)	0.02 (-0.02, 0.06)
**Group x Cycle x Cohort**		
Cohort C x Cycle 2		-0.02 (-0.06, 0.02)
Cohort D x Cycle 2		-0.05** (-0.08, -0.02)
Cohort C x T+		-0.01 (-0.06, 0.05)
Cohort D x T+		0.04 (-0.01, 0.10)
Cohort E x T+		0.03 (-0.02, 0.08)
Cohort A x T+		0.00 (-0.06, 0.07)
Cohort C x Cycle 2 x T+		0.01 (-0.04, 0.06)
Cohort D x Cycle 2 x T+		0.02 (-0.04, 0.07)
**Observations**	60,450	60,450

*p < 0.1; **p < 0.05; ***p < 0.01

Notes: (1) Controls for cohort; (2) Triple difference model with cohorts; Both specifications condition on number of sessions offered at the site during the given month

Specifications include strata FE

### Effect of ECD program on health and nutrition session attendance

We found no significant effect of offering ECD sessions to age-eligible children in communities on their monthly attendance at health nutrition sessions (Table 4), and no difference between T and T + in Cycle 2 (*p* = 0.385). Our results were robust to an array of robustness checks, including the inclusion of an additional two months in the pre-period, dropping ineligible sub-cohorts in each cycle, and the inclusion of population size as an additional control to account for imbalance. We found some heterogeneity in the treatment effect; children in sites in the Haute Matsiatra region (further from the capital city), with less educated CHWs, and in more remote sites (as represented by above-median distance to a healthcare center) had significantly lower attendance at health nutrition sessions in the T + group during Cycle 2 (Supplementary Fig. 5).

**Table 4:** Treatment effects on monthly attendance at health and nutrition sessions among children eligible for the ECD program

	(1)	(2)	(3)	(4)
**Group**				
Control	Ref.	Ref.	Ref.	Ref.
T	0.04 (-0.05, 0.13)	0.04 (-0.03, 0.12)	0.04 (-0.05, 0.13)	0.01 (-0.07, 0.10)
T+	0.09** (0.01, 0.18)	0.08* (0.00, 0.17)	0.09** (0.01, 0.18)	0.12** (0.05, 0.20)
**Cycle**				
Cycle 0	Ref.	Ref.	Ref.	Ref.
Cycle 1	0.03 (-0.01, 0.07)	0.06*** (0.03, 0.09)	0.03* (0.00, 0.07)	0.03 (-0.01, 0.07)
Cycle 2	0.04 (-0.01, 0.09)	0.06** (0.02, 0.11)	0.05* (-0.01, 0.10)	0.04 (-0.01, 0.09)
**Group x Cycle [DiD]**				
Group T x Cycle 1	0.01 (-0.04, 0.06)	0.01 (-0.03, 0.05)	0.01 (-0.04, 0.06)	0.01 (-0.04, 0.06)
Group T + x Cycle 1	-0.01 (-0.07, 0.05)	0.00 (-0.05, 0.06)	0.00 (-0.07, 0.06)	-0.01 (-0.07, 0.05)
Group T x Cycle 2	0.01 (-0.05, 0.08)	0.01 (-0.05, 0.07)	0.00 (-0.07, 0.08)	0.01 (-0.06, 0.08)
Group T + x Cycle 2	-0.02 (-0.10, 0.06)	-0.01 (-0.08, 0.06)	-0.03 (-0.11, 0.05)	-0.02 (-0.10, 0.06)
Target population size (per 10)				-0.02*** (-0.03, -0.01)
**Observations**	121,384	139,582	99,235	121,384
p-value for joint test (T)	0.878	0.912	0.952	0.886
p-value for joint test (T+)	0.838	0.854	0.635	0.824
p-value for T = T+ (Cycle 1)	0.492	0.817	0.684	0.481
p-value for T = T+ (Cycle 2)	0.385	0.578	0.362	0.379

*p < 0.1; **p < 0.05; ***p < 0.001

(1) 1 month of pre-intervention data included; (2) 3 months of pre-intervention data included; (3) Drops Cohort A in Cycle 1 and Cohort E in Cycle 2; (4) Controls for population size

Specifications includes strata and cohort FE

## Discussion

Our data provides support for the feasibility of adding ECD group sessions to existing health and nutrition services implemented by CHWs in rural Madagascar. In both intervention groups, CHWs delivered almost all sessions planned and children who attended the first session attended over three-quarters of sessions in the curriculum. There was also no effect of adding the ECD sessions on caregiver-child attendance at regular health and nutrition services, highlighting the finding that new services did not produce crowding-out effects. However, children from wealthier households, children who were already attending regular services, and children from less populated communities were more likely to register for ECD sessions. We also found that take-home toy boxes had no effect on monthly ECD session attendance but did promote children who did not attend any sessions during Cycle 1 to attend at least one session in Cycle 2. The goal of the government of Madagascar is to scale the ECD program to all sites involved in the PARN program to address child development alongside nutrition monitoring and other primary health services; several of our findings and limitations could be addressed for these future efforts, as well as in scale up efforts in other resource-constrained settings.

Overall, the MAHAY Mikolo program was feasible to integrate within the existing health and nutrition package delivered by frontline workers, as shown in the fidelity of the program, and was accepted by caregivers, as shown in the sustained attendance of children who signed up and attended without substituting away from other health services. Previous work analyzed outcomes of similar programs at scale in other contexts, including Bangladesh, Zambia, Kenya, Pakistan, and Lesotho, and also found that ECD programs were acceptable and feasible when integrated into the healthcare system and delivered through frontline workers [10–12, 21–23]. However, the number of spots in our program was limited by design and not proportional to the target population size in each site, and consequently, the program was not able to reach all age-eligible children. Additionally, we found that sites with a larger target population also had lower attendance in the regular health and nutrition activities (adjusting for site land area and thus, travel distances), even though there are no constraints on attendance capacity in the PARN program. Given that both the health and nutrition program and the ECD program are delivered by two CHWs regardless of target population size, government program coverage could be increased by increasing the frontline health system capacity. This may be difficult in this context given budgetary constraints but may be necessary prior to scaling up the program to additional communities [24]. Leveraging existing systems is cost-effective but requires investments in systems strengthening to deliver high-quality services and achieve universal coverage [8].

Another consideration for the scale-up of the program, especially in the context of limited coverage, is how to achieve penetration of ECD services, particularly to children most at risk for delayed development (such as children with severe growth faltering or from poorer households [25]). While implementing group-based sessions improves the efficiency of delivery on the supply side, the burden is shifted to caregivers to bring children to sessions, rather than CHWs reaching households. Poorer children who were not already attending health and nutrition sessions were less likely to register for the sessions, and, although we found no crowding out to the attendance at health and nutrition sessions, we also found no crowding in. This may reflect caregiver time or resource constraints, as CHWs reported that caregivers experienced multiple barriers to attending the group sessions related to time constraints (data not presented) or could potentially be a result of richer mothers being more receptive to new programs offered (an “early adopter” mentality). We also implemented this intervention during the pandemic, which could have had some effects on the delivery and attendance at group activities, especially in areas where populations were most vulnerable to economic shocks. In some sessions, children were accompanied by their adolescent siblings rather than caregivers. It may be useful in the context of rural, dispersed communities to consider an approach that combines group-based delivery with home-visit-based delivery and experiment with strategies to reach all households and maximize coverage [26], including the ones furthest away or most time-constrained. A combined approach was successfully implemented in Bangladesh and Kenya and cognitive measures were improved in combined group-based and home visits compared to control [10, 11]. If a combined approach is not feasible in the Madagascar context due to supply-side constraints, additional implementation strategies could be considered such as peer delivery or delivering sessions at the hamlet level, however, this would still require capacity strengthening. Another potential option in Madagascar could be to target children most at risk for delayed development, such as those lower on the socioeconomic status gradient [25] or experiencing stunting or underweight (assessed at nutrition sessions); a recent meta-analysis found heterogeneity by child nutrition status and larger intervention effects among programs that targeted undernourished children [27]. This could be effective, particularly in more populated communities where we found the lowest coverage, if supply-side constraints remain. Finally, training CHWs on effective mobilization strategies and outreach could also be considered to maximize take up and sustained attendance in the program.

We also found that the addition of take-home toy boxes and a toy and book library had no effect on monthly attendance at ECD sessions or health and nutrition sessions. Our hypothesis was that promoting behavior change through saliency of stimulation activities and the opportunity to rotate take-home toys and books would promote ECD program attendance and potential crowding in effects to other health services offered. However, continued attendance was high across both groups, independently of the extent to which toys or books were borrowed. Additionally, due to logistical constraints, we were unable to assess how development outcomes or aspects of the home caregiving environment (such as sustained play and implementation of recommendations around play) may have been affected by the toy boxes and libraries, beyond attendance. We found a small effect of toy boxes and libraries on ECD session attendance among children who did not attend any sessions during Cycle 1 (when toyboxes were not yet implemented in T+). It is possible that the demonstration effect of neighbors who had been exposed during Cycle 1 coupled with the possibility of receiving toy boxes and accessing the session materials enhanced the caregiver’s interest. Future work will include a review of qualitative data collected from caregivers to gain insights into these findings, such as reasons behind lack of take up or sustained attendance. Additional considerations for the feasibility of including toy boxes in the scale-up efforts are the feasibility of providing, managing, and maintaining the toy and book libraries; we did not collect information on this but plan to analyze qualitative data from CHW semi-structured interviews.

The ability to collect individual data through the administrative system was a strength of this study and continuing this ongoing measurement will be important for future evaluations of scale-up, including the feasibility and acceptability of the program in new communities [7]. Integrating new questions into the administrative modules (e.g. SES variables in the census or toy borrowing) proved difficult to implement due to the lack of remuneration for additional time-consuming, data-related tasks, resulting in high levels of missingness. However, the amount of administrative data captured from CHWs was essential to evaluating the impact of the program and providing policy recommendations. Aspects of future scale-up efforts, from training on new data modules, to continued program evaluation and improvement, can leverage these technological systems [28]. Additional considerations for capturing these important data include strategies to reduce the burden of data collection on already time-constrained frontline staff. For a period of our study, lack of payment to supervisors resulted in missing data. To resolve this, we leveraged novel methods, particularly OCR, as a feasible alternative for data entry. This technology may be able to reduce the time burden [29] and bypass a process where no regular training occurs. Lastly, a consideration for using administrative data was that we were unable to perform quality control and assurance on data collection at the CHW-level or data entry at the supervisor-level (this was also in part due to fieldwork restrictions during the pandemic).

Our study had some analytical limitations. We were unable to perform a random sampling of interested children for enrollment as specified in our protocol, however, this would not affect our treatment effect estimates given our site-level intention-to-treat analysis (which was prespecified). Additionally, there may have been some heterogeneity of treatment exposure over time due to variation in cycle start dates across sites, however, we assumed that this variation was balanced across treatment arms and would only result in non-differential misclassification and conservative estimates. We also began the study during the pandemic and over one year prior to the implementation of the census and the start of the intervention, which may have caused the imbalance in population size, however, we used a differences-in-differences estimator to estimate the treatment effect. Finally, we did not assess child development outcomes that the program aims to address or more proximal outcomes such as measures of the home caregiving environment, but this was by design as our study goal was to analyze process outcomes and pathways to scaling.

## Conclusion

In conclusion, we found that integrating group sessions focusing on ECD into regular health and nutrition programming in a low-income context is feasible. There is no clear evidence that expanding the scope of the health and nutrition program affects the take up of regular activities offered. However, investment in health services to produce equitable coverage in more populated areas is needed to reach all children, especially those not already taking part in health and nutrition services who may be at risk for delayed development.

## Electronic supplementary material

Below is the link to the electronic supplementary material.

Supplementary Material 1

## Data Availability

Deidentified administrative data are available upon reasonable request for scientific inquiry. Please contact Emanuela Galasso (egalasso@worldbank.org).
