# Supplementary material for: Integrating early child development into an existing health and nutrition program: evidence from a cluster-randomized controlled trial
Source: BMC Public Health. 2024 Sep 27;24:2583. doi: 10.1186/s12889-024-20149-w (PMC11428953; doi:10.1186/s12889-024-20149-w)
Supplement: Supplementary file 1 — Supplementary Material 1 [file 12889_2024_20149_MOESM1_ESM.docx]

Supplementary Figure 1. Duration of exposure and eligibility by cohort, age, and study cycle


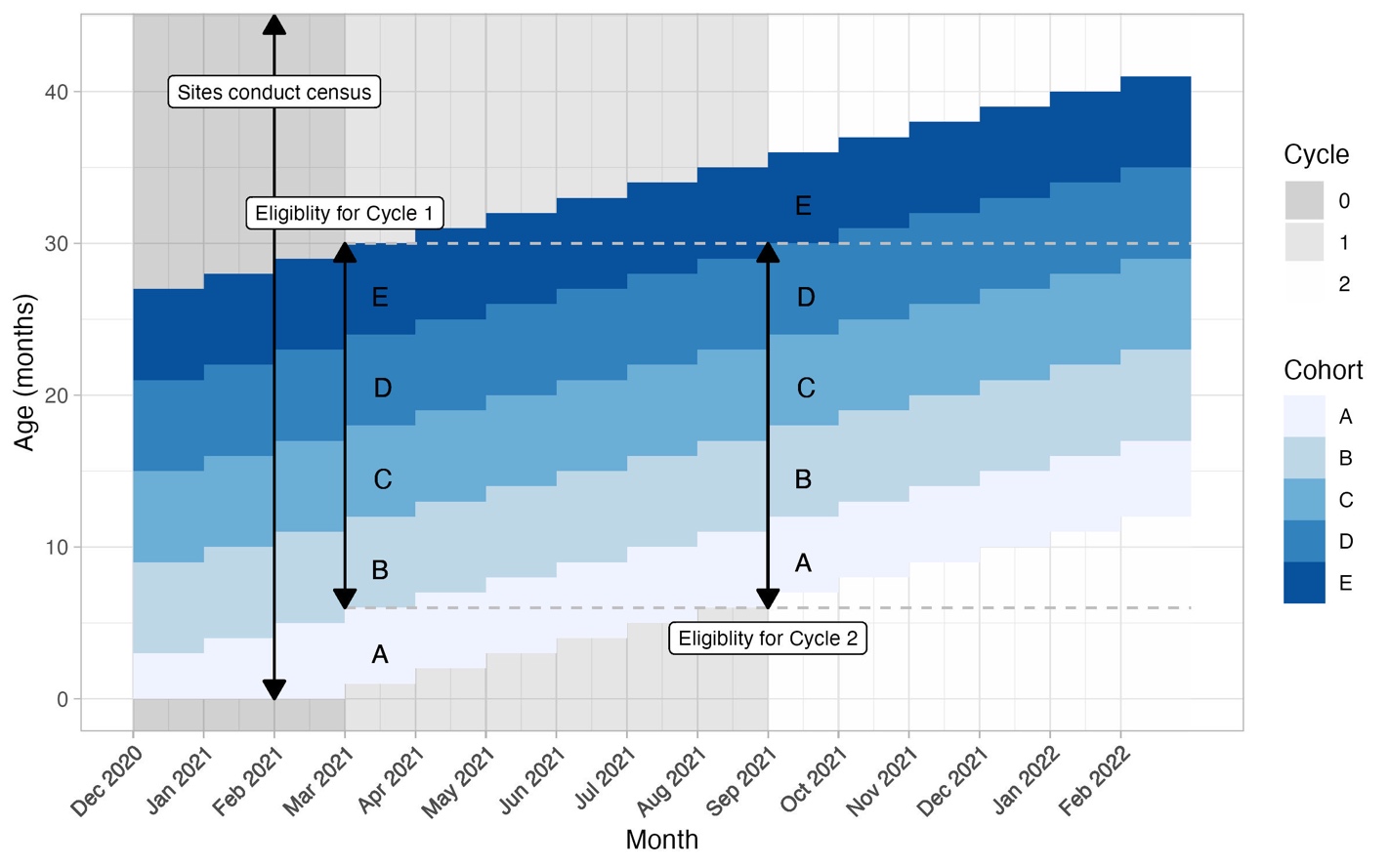


Note: Ages within gray dashed lines indicate eligibility for ECD group sessions. Cohorts were defined as age group at the beginning of cycle 1 ( A = 0 to <6 months, B = 6 to <12 months, C = 12 to <18 months, D = 18 to <24 months, E = 24 to <30 months).

Supplementary Figure 2. CONSORT study flow diagram


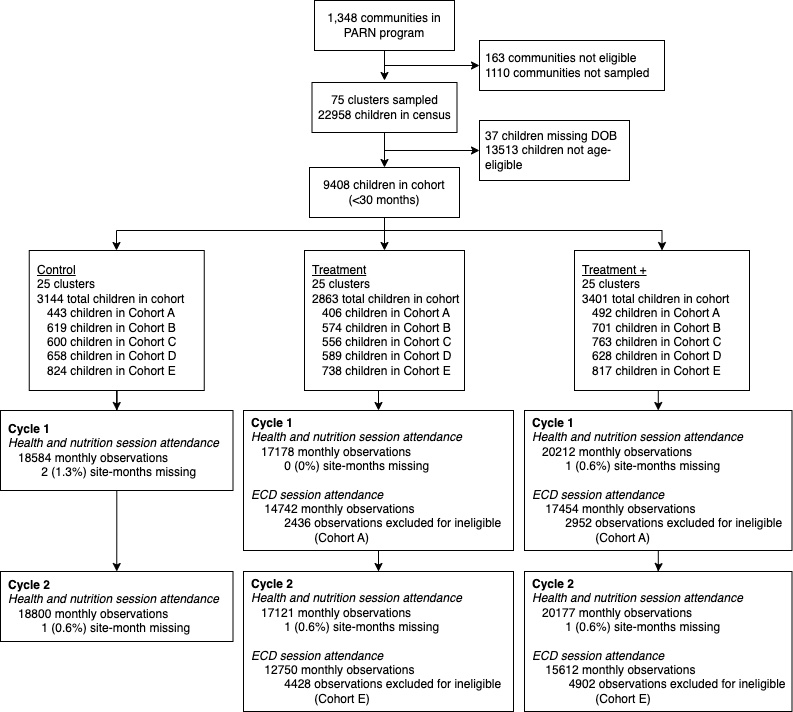


Supplementary Figure 3. Pre-trends for health and nutrition session attendance


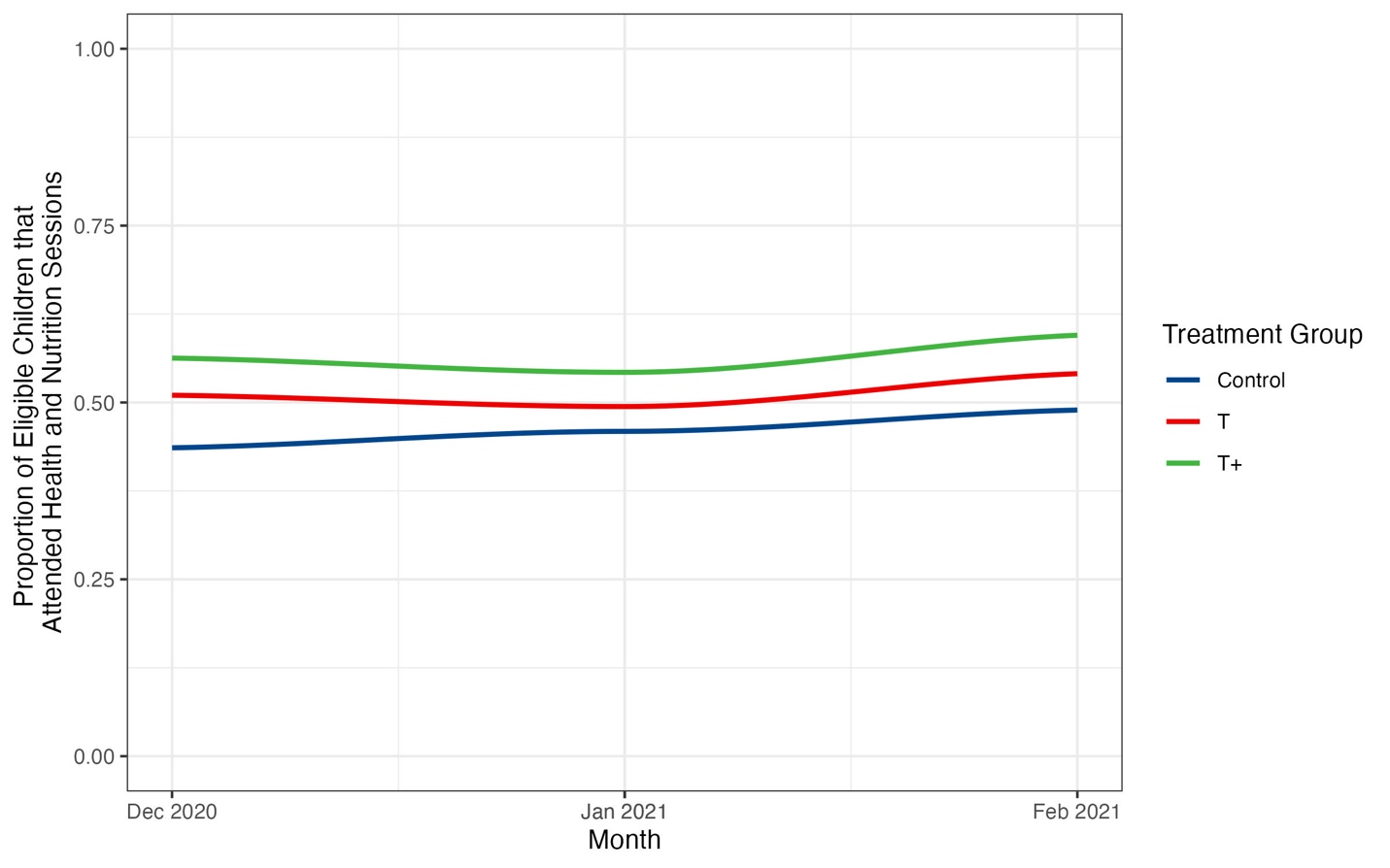


Note: Lines are averaged across site-level averages among age-eligible children.

Supplementary Figure 4. Ages of children that attended each age group, by cycle


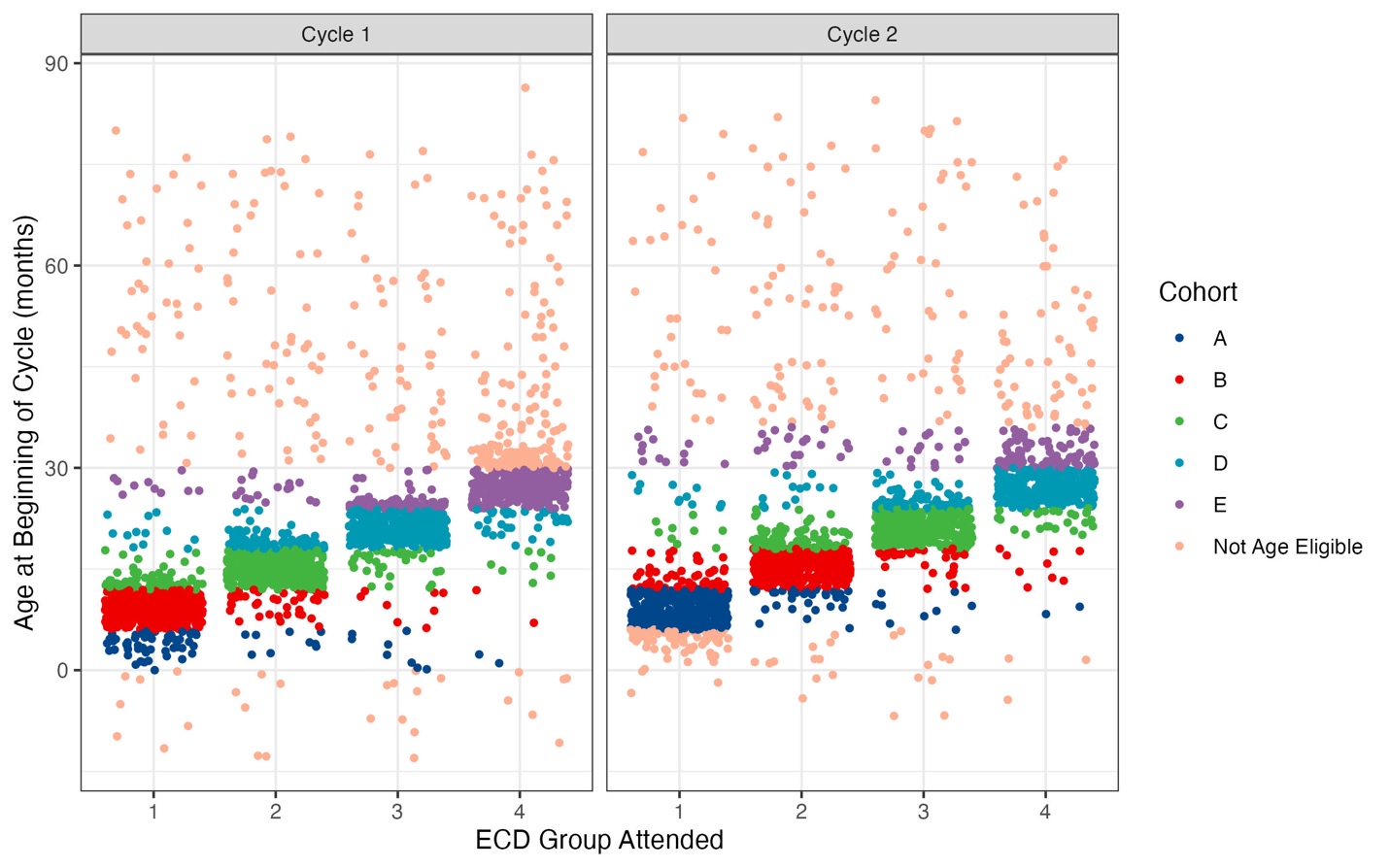


Note: Groups are targeted to the following ages: 1 - 6 to <12 months, 2 - 12 to <18 months, 3 - 18 to <24 months, 4 - 24 to <30 months

**Supplementary Table 1. Variables associated with signing up for ECD sessions, among age-eligible children**

|  | Null model | Strata variables | Individual variables | +SES |
| --- | --- | --- | --- | --- |
| Distance from CSB (km) |  | 0.00 (-0.02, 0.01) | 0.00 (-0.01, 0.01) | 0.01 (-0.01, 0.03) |
| Target population size (per 10) |  | -0.03*** (-0.04, -0.03) | -0.03*** (-0.04, -0.02) | -0.03*** (-0.04, -0.02) |
| **Region** |  |  |  |  |
| Haute Matsiatra |  | - | - | - |
| Amoron’i Mania |  | 0.02 (-0.04, 0.07) | 0.06** (0.02, 0.10) | 0.12** (0.05, 0.19) |
| **CHW education** |  |  |  |  |
| Neither CHW has Secondary II  education |  | - | - | - |
| At least 1 CHW has Secondary II  education |  | 0.01 (-0.05, 0.06) | 0.04* (-0.01, 0.08) | 0.03 (-0.04, 0.10) |
| **Cycle** |  |  |  |  |
| Cycle 1 |  | - | - | - |
| Cycle 2 |  | 0.02** (0.00, 0.04) | -0.01 (-0.03, 0.02) | 0.00 (-0.05, 0.04) |
| **Group** |  |  |  |  |
| T |  | - | - | - |
| T+ |  | 0.01 (-0.05, 0.06) | -0.01 (-0.05, 0.03) | -0.01 (-0.08, 0.06) |
| Cycle 2 x Group T+ |  | -0.02 (-0.05, 0.01) | -0.02 (-0.05, 0.01) | -0.01 (-0.06, 0.05) |
| **Child gender** |  |  |  |  |
| Female |  |  |  |  |
| Male |  |  | 0.01 (-0.01, 0.02) | 0.01 (-0.02, 0.05) |
| **Cohort** |  |  |  |  |
| B |  |  | - | - |
| C |  |  | 0.00 (-0.03, 0.02) | 0.01 (-0.04, 0.07) |
| D |  |  | 0.01 (-0.01, 0.04) | 0.04 (-0.01, 0.09) |
| E |  |  | -0.05** (-0.08, -0.02) | -0.01 (-0.06, 0.04) |
| A |  |  | 0.02 (-0.01, 0.06) | 0.05 (-0.04, 0.14) |
| Number of siblings <2 years |  |  | 0.02 (-0.03, 0.06) | 0.05 (-0.03, 0.13) |
| Number of siblings 2-5 years |  |  | -0.01 (-0.04, 0.01) | -0.03* (-0.07, 0.00) |
| Number of siblings >5 years |  |  | 0.01 (-0.03, 0.04) | 0.01 (-0.04, 0.06) |
| Number of health and nutrition sessions  attended in past 6 months |  |  | 0.06*** (0.05, 0.06) | 0.08*** (0.07, 0.09) |
| Maternal education (years) |  |  |  | 0.00 (-0.01, 0.01) |
| **Wall material** |  |  |  |  |
| Stem, bark, leaf, plant |  |  |  | - |
| Clay, brick, unfired sheet metal |  |  |  | 0.08** (0.00, 0.16) |
| Baked brick, cinder block, stone |  |  |  | 0.12** (0.03, 0.22) |
| **Roof material** |  |  |  |  |
| Stem, bark, leaf, plant |  |  |  | - |
| Tile, sheet metal, concrete, other |  |  |  | 0.00 (-0.04, 0.04) |
| (Intercept) | 0.35*** (0.31, 0.39) | 0.65*** (0.55, 0.74) | 0.43*** (0.35, 0.51) | 0.29*** (0.14, 0.44) |
| *𝚿3* | *0.28* | *0.28* | *0.25* | *0.23* |
| *𝚿2* | *0.14* | *0.09* | *0.07* | *0.10* |
| *𝛔* | *0.34* | *0.34* | *0.34* | *0.36* |
| Observations | 10075 | 10075 | 10075 | 3279 |

*p<0.1; **p<0.05; ***p<0.01

Notes: Standard errors are clustered at the site level. Analysis restricted to T and T+ sites.

Cohorts were defined as age group at the beginning of cycle 1 ( A = 0 to <6 months, B = 6 to <12 months,

C = 12 to <18 months, D = 18 to <24 months, E = 24 to <30 months).

*𝚿3* is the standard deviation of the site-level random intercepts, *𝚿2* is the standard deviation of the child-level random

intercepts, and *𝛔* is the standard deviation of the residual error.

Supplementary Table 2. Missingness of SES variables (wall, roof, maternal education) among children included in registration analysis (eligible for Cycle 1 or Cycle 2 in T and T+ groups).

|  | **Missing data on SES variables** |
| --- | --- |
| Group |  |
| T | - |
| T+ | -0.10** (-0.17, -0.02) |
| Target population size (per 10) | 0.00 (-0.01, 0.02) |
| Cohort |  |
| A | - |
| B | -0.12*** (-0.17, -0.07) |
| C | -0.15*** (-0.22, -0.08) |
| D | -0.22*** (-0.29, -0.15) |
| E | -0.46*** (-0.52, -0.39) |
| Observations | 6264 |
| *p<0.1; **p<0.05; ***p<0.001  Notes: Specification includes strata FE. Cohorts were defined by age group at the beginning of Cycle 1 ( A = 0 to <6 months, B = 6 to <12 months, C = 12 to <18 months, D = 18 to <24 months, E = 24 to <30 months). | |

Supplementary Table 3. Effect of T+ on the probability of parents accompanying children to ECD sessions

|  | **Attendance by parents** |
| --- | --- |
| Group |  |
| T | - |
| T+ | 0.00  (-0.05, 0.05) |
| Cycle |  |
| Cycle 1 | - |
| Cycle 2 | -0.02  (-0.07, 0.03) |
| Cycle 2 x Group T+ | -0.02  (-0.07, 0.02) |
| ECD Group Attended |  |
| 1 | - |
| 2 | -0.04***  (-0.06, -0.02) |
| 3 | -0.07***  (-0.09, -0.04) |
| 4 | -0.09***  (-0.12, -0.06) |
| Observations | 32259 |
| *p<0.1; **p<0.05; ***p<0.01  Notes: Specification includes strata FE. Groups are targeted to the following ages: 1 - 6-<12 months, 2 - 12-<18 months, 3 - 18-<24 months, 4 - 24-<30 months. Includes sample of children who attended. | |

Supplementary Table 4. Effects of T+ on any ECD session attendance in Cycle 2

|  | **Among children who attended Cycle 1** | **Among children who did not attend Cycle 1** |
| --- | --- | --- |
| T+ | 0.02  (-0.05, 0.10) | 0.04**  (0.00, 0.08) |
| Cohort C | 0.05  (-0.01, 0.11) | 0.00  (-0.03, 0.04) |
| Cohort B | 0.03  (-0.04, 0.09) | 0.06**  (0.02, 0.09) |
| Target population size (per 10) | -0.03***  (-0.04, -0.01) | -0.01**  (-0.02, 0.00) |
| Observations | 1469 | 2342 |
| *p<0.1; **p<0.05; ***p<0.01  Notes: (1) Among children that were eligible and attended any sessions during Cycle 1; (2) Among children that were eligible and never attended any sessions during Cycle 1. Specification includes strata FE. | | |

Supplementary Figure 5. Heterogeneity of treatment effects on health and nutrition session attendance using stratifying variables


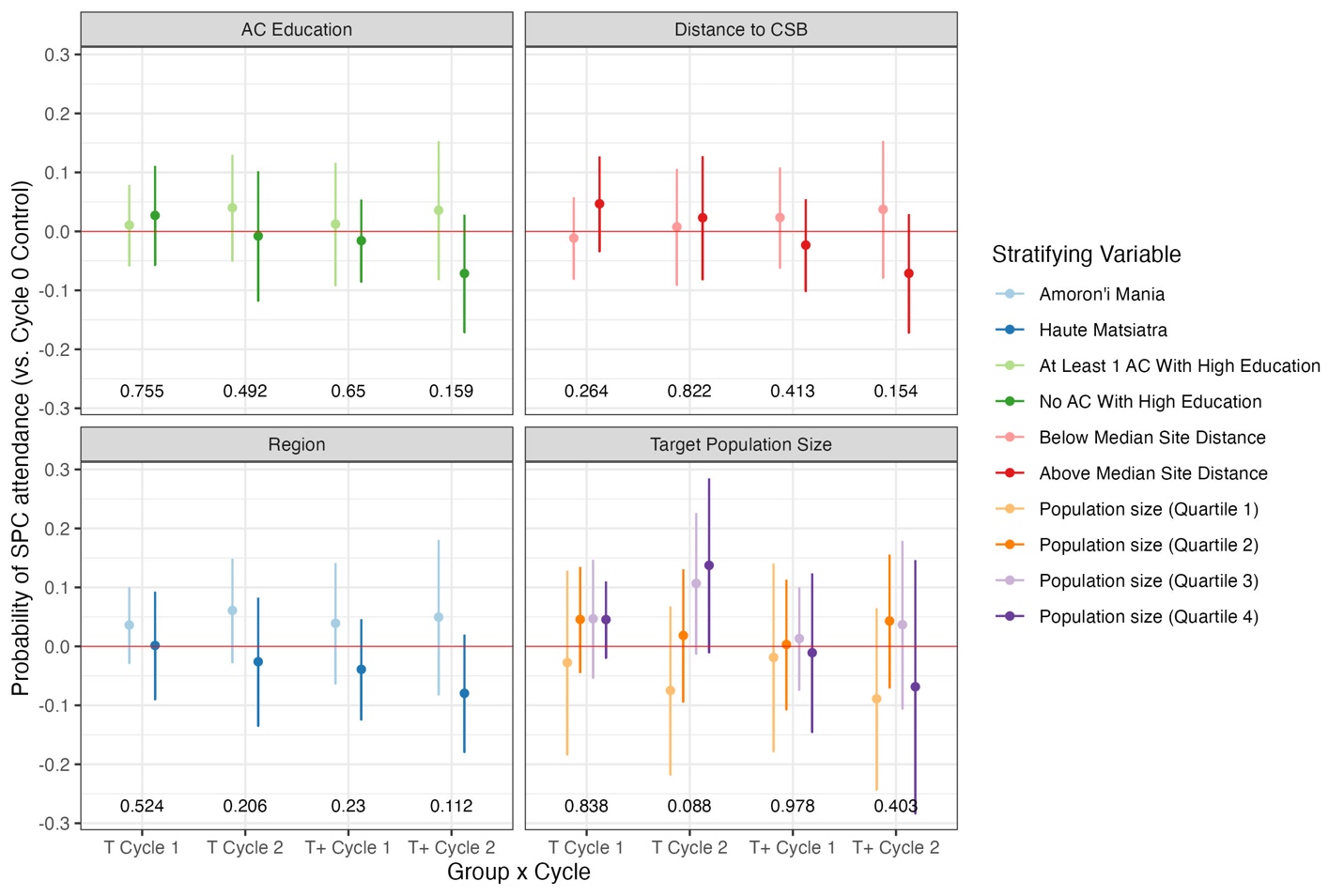


Note: Labels are p-values of the three-way interaction coefficient (or set of coefficients) in a triple difference model.
